# Immunotherapeutic Potential of Extracellular Vesicles

**DOI:** 10.3389/fimmu.2014.00518

**Published:** 2014-10-22

**Authors:** Bin Zhang, Yijun Yin, Ruenn Chai Lai, Sai Kiang Lim

**Affiliations:** ^1^Exosome and Secreted Nano-vesicle Group, A*STAR Institute of Medical Biology, Singapore; ^2^Department of Surgery, Yong Loo Lin School of Medicine, National University of Singapore, Singapore

**Keywords:** extracellular vesicles, exosomes, immunomodulation, innate immunity, adaptive immunity, immunotherapy

## Abstract

Extracellular vesicle or EV is a term that encompasses all classes of secreted lipid membrane vesicles. Despite being scientific novelties, EVs are gaining importance as a mediator of important physiological and pathological intercellular activities possibly through the transfer of their cargo of protein and RNA between cells. In particular, exosomes, the currently best characterized EVs have been notable for their *in vitro* and *in vivo* immunomodulatory activities. Exosomes are nanometer-sized endosome-derived vesicles secreted by many cell types and their immunomodulatory potential is independent of their cell source. Besides immune cells such as dendritic cells, macrophages, and T cells, cancer and stem cells also secrete immunologically active exosomes that could influence both physiological and pathological processes. The immunological activities of exosomes affect both innate and adaptive immunity and include antigen presentation, T cell activation, T cell polarization to regulatory T cells, immune suppression, and anti-inflammation. As such, exosomes carry much immunotherapeutic potential as a therapeutic agent and a therapeutic target.

## Introduction

Extracellular vesicles (EVs) are increasingly implicated as a major mode of intercellular communication. Most cell types are known to secrete EVs which are essentially bi-lipid membrane vesicles carrying a complex cargo of proteins and RNAs. These EVs can be taken up by other cell types thereby transferring proteins and RNAs from one cell to another.

There are many classes of EVs such as exosomes, microvesicles, ectosomes, membrane particles, exosome-like vesicles and apoptotic bodies, and they could be distinguished by their biogenesis pathway, size, flotation density on a sucrose gradient, lipid composition, sedimentation force, and protein cargo [reviewed in Ref. (1, 2)]. However, many of these differentiating parameters such as size, flotation density on a sucrose gradient, lipid composition, sedimentation force, and protein cargo are not discrete values that are exclusive to a specific class of EVs. Consequently, classification of EVs has been challenging. Exosomes are presently the best characterized EVs. They are defined as membrane vesicles of 50–100 nm diameter containing proteins, RNAs, and lipids (3–8). They are secreted by cells when endosomal membranes invaginate inward to form multivesicular bodies (MVBs) and the MVs fused with the plasma membrane. This endosomal biogenesis is a distinctive feature of exosomes, and is presently known to be unique to exosomes and not any of the other classes of EVs. As endocytosis is most active at specialized microdomains in plasma membrane such as lipid rafts, exosomes such as mesenchymal stem cell (MSC) exosomes have membranes enriched in elements of lipid rafts such as GM1 gangliosides and transferrin receptors (9). However, ascertaining the biogenesis of EVs is technically challenging and not always practical, and consequently, the term “exosomes” have been used generically to describe any EVs that share some of the biophysical or biochemical parameters of exosomes without validating their biogenesis. Hence, the term EVs and exosomes in this review will be used synonymously.

Many cell types are known to secrete EVs, and they include epithelial cells (10, 11), fibroblasts (12), erythrocytes (13, 14), platelets (15), mast cells (16), tumor cells (17–19), stem cells (20–22), and immune cells such as dendritic cells (DCs) (23, 24), monocytes (25, 26), macrophages (27, 28), NK cells (29, 30), B lymphocytes (31, 32), and T lymphocytes (33, 34). However, the physiological functions of EVs remain tenuous. Part of this could be attributed to the lack of a definitive criterion to purify, characterize, and classify the classes of EVs unambiguously.

Exosomes were first thought to be a cellular means for the disposal of redundant proteins by groups studying reticulocyte maturation (35–37), but they are now generally viewed as mediators of intercellular communication through the transfer of biologically active materials. However, with the present lack of clarity in defining the classes of EVs, the role of EVs or exosomes as mediators of intercellular communication, and their effects on biological or physiological processes remains a conundrum. For example, do cells secrete or even have the capacity to produce all classes of EVs? Do cells secrete more than one class of EVs at any one time? Are secretion or production of EVs and their cargo regulated? It is likely that our present day understanding of EV functions is an amalgam of the diametrically different functions of different EV classes, leading to a confusing and sometimes contradictory perception of EV or exosome functions. One of the earliest reported physiological targets of exosome-mediated intercellular communication is the immune system. B lymphocytes were the first immune cell type reported to secrete vesicles and these vesicles express abundant major histocompatibility complex (MHC) Class I and II molecules, B7.1 (CD80) and B7.2 (CD86) co-stimulatory molecules, and ICAM-1 (CD54) adhesion molecules. Consistent with the presence of these proteins, which are important in antigen presentation, the vesicles could activate CD4^+^ T cells suggesting that they mediate antigen presentation by lymphocytes (38). Subsequently, other immune cells such as DCs and mast cells were also found to secrete EVs/exosomes (39, 40). Incidentally, non-immune cells have also been reported to modulate the immune system by exosomes. For example, cancer cells are known to modulate the immune system to facilitate growth and metastasis. One of the earliest reports described the downregulation of surface NKG2D expression on NK cells and CD8^+^ T cells by exosomes from cancer cells or mesothelioma patients (41). We have also demonstrated that exosomes from MSCs modulate the immune system through TLRs and induce expansion of regulatory T cells (Tregs) (22). Together, these studies demonstrate the immense immunotherapeutic potential of exosomes produced by both immune and non-immune cells and also highlight the divergent effects of EVs/exosomes on the immune system. While this divergence could be due to a difference in cell source of the EVs/exosomes, other parameters such as the classes of EVs and the methods of isolation are equally important.

## Biogenesis and Preparation of EVs

**Figure 1:**
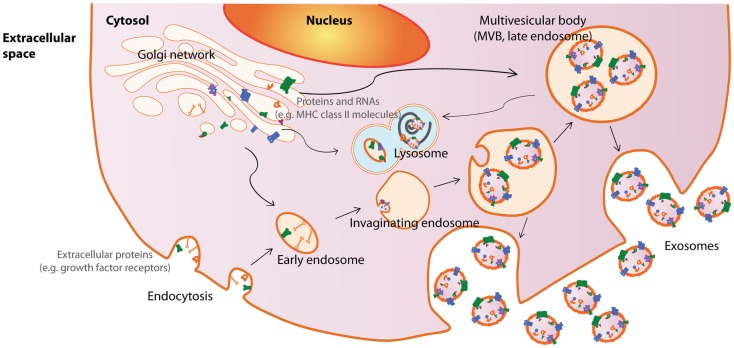
**Exosome biogenesis and secretion**. Exosomes are released by cells when intracellular organelles called multivesicular bodies (MVBs) fuse with the plasma membrane. MVBs are formed by invaginations of late endosomes, which contained molecules from the Golgi (e.g., MHC class II molecules) or the cell surface (e.g., growth factor receptors). Consequently, exosomes contain cytosolic materials and are enriched in endosome-associated protein markers such as the the Rab proteins, ALIX, TSG101, and MHC class II molecules or endocytic proteins, such as transferrin receptors and clathrins. This figure was modified from Lai et al. (169).

Among the EVs, exosomes are best defined in terms of their biogenesis and consequently, are the best characterized EVs. Exosomes are small membrane vesicles about 50–100 nm in diameter that are secreted by cells into the extracellular compartment when MVBs in the cells fuse with the plasma membrane [reviewed in Ref. (42)]. MVBs in turn are formed by membrane invagination of late endosomes. Therefore, all cells that could generate MVBs have the potential to produce exosomes. As the biogenesis of exosomes from endosomes involves a reverse budding process, exosome membranes have the same directional orientation as cells with the cytosol inside the luminal space, and extracellular domains of membrane proteins exposed at external surface. The release of exosomes through fusion of MVBs with the plasma membrane has been documented by electron microscopy while the endosomal origin of exosomes are supported by the enrichment of endosome-associated proteins such as the Rab proteins, ALIX, TSG101, or endocytic proteins such as transferring receptors, clathrins (3, 9, 43, 44) (Figure 1).

Since EVs are released to the extracellular space, these vesicles are routinely purified from cell culture supernatants or biological fluids. EV purification from cell-conditioned culture media classically involves a series of differential centrifugations to remove dead cells and large debris, and then a final high speed ultracentrifugation of about 100,000 × *g* for at least 1 h to pellet EVs (37, 45, 46). As exosomes have a characteristic flotation density of 1.13–1.21 g/ml, an additional equilibrium density gradient centrifugation is often used to enhance the purity of the exosome preparation. However, ultracentrifugation has poor scalability and is thus not amenable to large scale manufacturing processes. Another common method is to isolate EVs or exosomes by their size as in size exclusion chromatography (47, 48). To enhance the resolution of size exclusion chromatography, our laboratory routinely uses high-performance liquid chromatography (HPLC) to purify exosomes from MSC-conditioned medium (49). Other methods of isolation include immunoaffinity chromatography where exosomes are captured using antibody that recognizes a marker enriched on exosomes such as MHC class II molecules for antigen-presenting cells (APC)-derived exosomes (50), A33 (5), or EpCAM (51) for exosomes from tumor cell line, or CD63, which could theoretically be used for exosomes from all sources (52, 53). However, these isolation techniques frequently require biologically harsh conditions, such as low pH or high salt concentration, to release the bound EVs such that the biological activity of the EVs would be compromised. Like the other isolation techniques, these methods may not differentiate between different classes of EVs or between EVs and large protein complexes. Despite the challenges in isolating specific classes of EVs, the isolation of exosomes for use in clinical testing has already been described (54, 55). The isolation process usually includes a filtration step to isolate large complexes followed by equilibrium density gradient centrifugation to isolate complexes according to density.

## EVs and Immunity

Vertebrates have two arms of immune systems, namely the innate and adaptive immune systems. The innate immune system is an evolutionarily conserved immune system found in all multi-cellular organisms while the adaptive immune system is found only in vertebrates (56). The innate immune system is generally the first line of defense against microbial pathogens or tissue damage and mediates inflammation. It is activated through a limited set of germline-encoded receptors that recognizes pathogen-associated molecular patterns (PAMPs) from infectious agents, or damage-associated molecular patterns (DAMPs) releasing from dying cells (57, 58). These germline-encoded receptors are often referred to as pattern recognition receptors (PRRs). In mammals, Toll-like receptors (TLRs) are the best characterized examples. In addition, other receptors such as nucleotide-binding and oligomerization domain (NOD)-like receptors (NLRs), retinoic acid-inducible gene I (RIG-I)-like receptors (RLRs), and some C-type lectin receptors (CLRs) also recognize specific components of microbes and are therefore considered as innate immune receptors (59, 60). In contrast, antigen receptors in the adaptive immune system are not germline-encoded but generated through somatic hypermutations (61). Hence the adaptive immune system has an immensely wider repertoire of antigen receptors compared to the limited set of receptors for PAMPs or DAMPs in the innate immune system. As such, the adaptive immune system also has the potential to fine tune its antigen recognition to a high degree of specificity and avidity. Induction of adaptive immunity depends on antigen recognition by antigen receptors on adaptive immune cells followed by subsequent activation and clonal expansion of cells carrying the appropriate antigen-specific receptors (62). The two main cell types in adaptive immune system are T and B cells. Unlike B cells, naïve T cells do not recognize antigens and their capacity to recognize antigens have to be activated through a process known as antigen presentation. During antigen presentation, APCs such as DCs or macrophages internalize foreign antigens, process, and load the processed antigens onto MHC I and MHC II molecules for presentation to naïve CD8^+^ and CD4^+^ T cells, respectively. Together with co-stimulatory molecules such as CD80 and CD86, the antigen-MHC class I or II complex on APCs activates T cells and imparts the memory of the antigen to the T cells (63–66).

**Figure 2:**
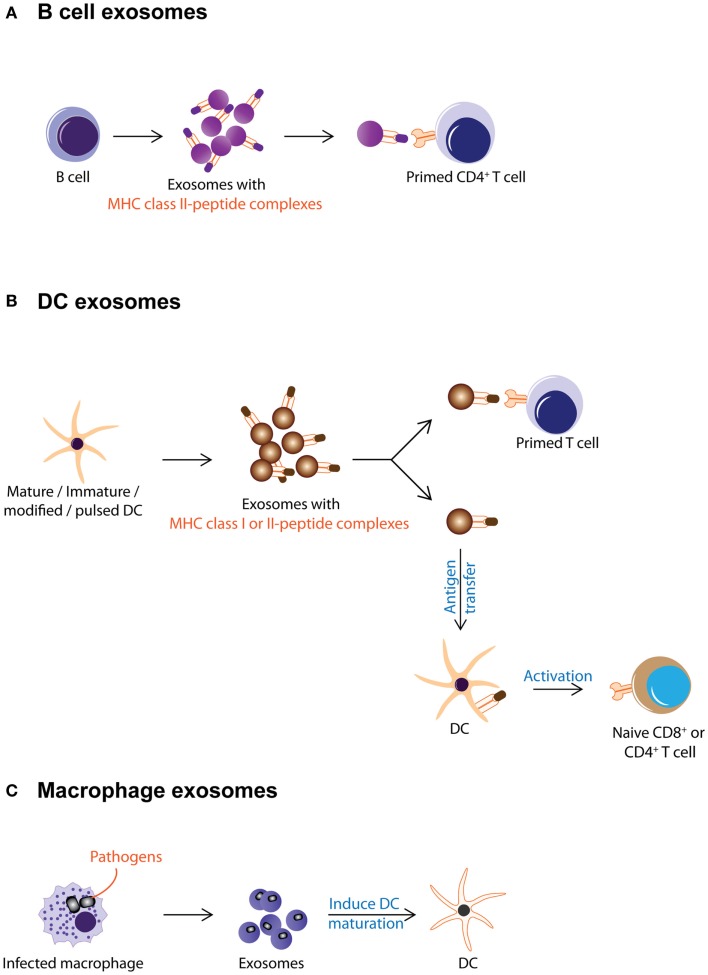
**Exosomes from immune cells are shown**. This figure summarizes the different known activities of exosomes from immune cells. **(A)** B cell secretes exosomes carrying MHC class II–peptide complexes as a mode of antigen presentation to primed CD4^+^ T cell. **(B)** DC-derived exosomes carry MHC class I or II-peptide complexes that can be either directly recognized by pre-activated CD4^+^ or CD8^+^ T cells or captured and presented by DCs to activate naïve T cells. **(C)** Macrophages infected with pathogens secrete exosomes with pathogen antigens. These exosomes induce maturation of DCs and promote secretion of pro-inflammatory cytokines.

**Figure 3:**
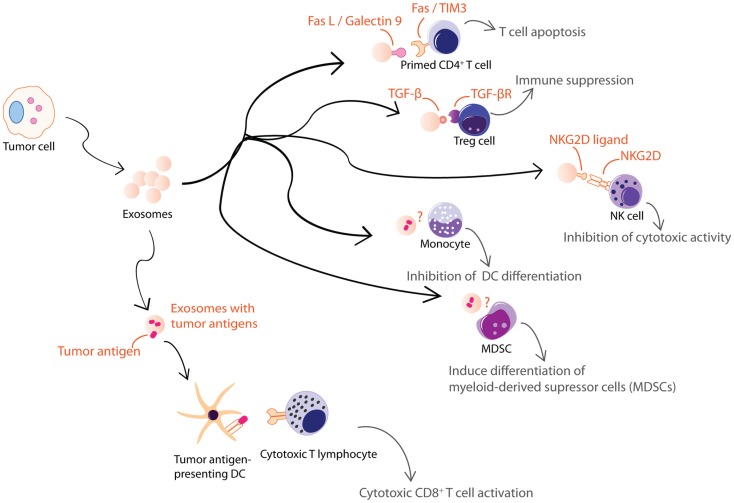
**Exosomes from tumor cells are shown**. Tumor cell-derived exosomes can be either immune suppressive or activating. These exosomes exert some of their immune suppressive activities on T cells through FasL, galectin-9, TGFβ, or NKG2D ligand carried by the exosomes. Others inhibit differentiation of DCs or MDSCs through unknown mechanisms. However, tumor cell-derived exosomes also carry tumor antigens that could elicit an anti-tumor response.

Antigen presentation was generally assumed to be a cell–cell interaction until it was discovered that Epstein–Barr virus-transformed B lymphocytes secreted exosomes carrying antigenic peptide bound-MHC class II dimers. These exosomes could induce antigen-specific MHC class II-restricted T cell response (Figure 2A) (38). Subsequently, DCs were also found to secrete exosomes with MHC class I and II, and T cell co-stimulatory molecules. When pulsed with tumor peptides, DCs secrete exosomes that could activate cytotoxic T lymphocytes and suppress tumor growth in a T cell-dependent manner (Figure 2B) (23). It was further observed that allogeneic and autologous exosomes were equally efficient in eliciting anti-tumor protection (67). Since then, exosomes have been reported to play an extensive role in antigen presentation. Exosomes purified from ascites of tumor patients (68) or culture medium of tumor cell lines (17, 69) have been shown to carry tumor antigens. When pulsed with these exosomes, DCs endocytose the exosomes and present the exosome-associated tumor antigens on their MHC molecules to activate tumor-specific cytotoxic T lymphocytes (Figure 3) (17, 69–71). However, the role of exosomes in antigen presentation is not restricted to the conveyance of antigens to APCs for T cell activation. Some exosomes, particularly those derived from mature DCs are known to carry MHC II–peptide complexes and they could activate T lymphocytes directly (Figure 2B). For example, DC-derived exosomes could activate CD8^+^ cytotoxic T lymphocytes clones by themselves (72–74). However, it has been reported that such activation is generally inefficient compared to activation in the presence of DCs (75).

The antigen-presenting capacity of exosomes from DCs depends on the maturation state of the DCs with less exosomes being produced as the cells mature (76–79). Unlike exosomes from immature DCs, exosomes from mature DCs carry MHC class II and co-stimulatory molecules such as B7.2, ICAM-1, but not MFG-E8 on their membrane (70, 80–84). As such, they can interact directly with T cells to activate the immune system and eradicate tumor or bacteria or virus (23, 85, 86). However, these MHC class II-expressing exosomes have also been implicated in immune suppression and prolonged survival of allografts. When administered to rats prior to heart allograft transplantation, MHC class II-expressing exosomes from donor DCs prolong graft survival with a significant concomitant decrease in recipient CD4^+^ T cells and increased anti-donor MHC class II alloantibody production suggesting that these exosomes can modulate immune response by either inducing tolerance or immune reactivity (Figure 2B) (87).

On the other hand, exosomes from immature DCs while produced in greater abundance, are 50- to 100-fold less potent in T cell stimulation (82). This attenuated potency has been attributed to the absence of MHC class II and co-stimulatory molecules such as B7.2, ICAM-1 on their membrane (70, 80–84). Instead of T cell stimulation, these exosomes help distribute antigens to other APCs for antigen presentation and subsequent immune modulation (Figure 2B) (79). Additionally, exosomes from immature DCs carry MFG-E8, which binds phosphatidylserine and opsonizes apoptotic cells for phagocytosis (88), possibly enhancing the antigen presentation function of immature DC exosomes.

Exosomes secreted by immature DCs could be either immune suppressive or activating. Exosomes derived from immature, gene-modified, or interleukin (IL)-10-treated DCs have been shown to induce immune suppression in murine models of transplantation and auto-immune disease (89–97). At the same time, when immature DCs were pulsed with pathogen-associated antigens such as *Toxoplasma gondii* antigen, diphtheria toxin, or *Eimeria tenella* antigens, they produce exosomes that confer immune protection against these pathogens (23, 67, 85, 86, 98–100). Incidentally, this immune protection is known to include polyclonal activation of B cells as well (101). Thus exosomes from immature DCs elicit the immune suppression or immune activation (Figure 2B) [reviewed in Ref. (90, 102, 103)].

This capacity of DC-derived exosome to suppress or activate the immune system indicates that exosome is an important component of the complex immune network in mediating a steady state between immune tolerance and rejection. It also serves an underpinning rationale for the use of DC-derived exosomes in vaccination trials to treat melanoma and non-small cell lung cancer patients (102).

Besides DCs, which secrete exosomes targeting mainly the adaptive immunity (104), other cell types when challenged also secrete immunologically active exosomes targeting primarily but not exclusively the innate immunity. Macrophages when infected by pathogens such as mycobacterium or toxoplasma, release EVs containing PAMPs that induce secretion of pro-inflammatory cytokines and recruitment of immune cells (Figure 2C) (105–107). These EVs have also been implicated in the progression of auto-immune diseases. For example, it was observed that fibroblasts from synovial fluid of patients with rheumatoid arthritis secreted EVs carrying active membrane-bound TNF-α can inhibit T cell activation-induced cell death (AICD) (108) and EVs present in the bronchoalveolar fluid of patients with sarcoidosis also displayed pro-inflammatory activities (109).

Tumor cells also secrete immunologically active EVs. Some of these EVs are pro-inflammatory while others are immune suppressive (Figure 3). Heat-shocked tumor cells secrete EVs that promote NK cell activity (110), secretion of TNF-α by macrophages (111), and stimulate T cell activation (112, 113). Anticancer drug-treated carcinoma cells release exosomes with heat shock proteins (HSPs) that elicit effective NK cell anti-tumor responses (114) while exosomes from ascites of gastric cancer patients induce a tumor-specific CTL response (115). On the other hand, EVs from tumor cell lines or tumor-bearing patients were found to carry various immunosuppressive molecules that when tested *in vitro*, induced T cell apoptosis via Fas ligand (FasL) (48, 116–118) and galectin-9 (119, 120), inhibited IL-2-induced T cell proliferation (121), and/or promote differentiation into Tregs (122, 123). They also decreased NK cell activity (41, 124, 125), inhibited myeloid precursor differentiation into DCs (126), and promoted the differentiation of myeloid-derived supressor cells (MDSCs) (127–130). Hence, tumor cells secrete EVs that could both inhibit tumor growth by eliciting anti-tumor immune responses (131) and promote tumor growth by inhibiting anti-tumor immunity or enhancing angiogenesis and/or metastases (132).

**Figure 4:**
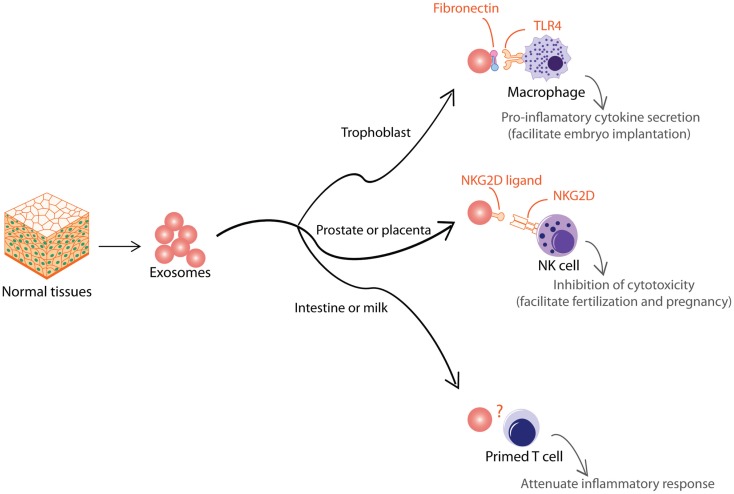
**Exosomes from normal tissues are shown**. Normal tissue-derived exosomes exert multiple immune-modulatory activities to support normal physiological processes such as embryo implantation, fertilization, pregnancy, and oral tolerance.

Aside from pathological situations, exosomes are also used to modulate immune activity to support normal physiological processes. Like tumor EVs, normal tissues also produce EVs that are functionally dichotomous (Figure 4). During early pregnancy, trophoblast secretes exosomes to recruit and educate monocytes to initiate a pro-inflammatory microenvironment essential for embryo implantation, angiogenesis, and stromal remodeling associated with early pregnancy (133). These exosomes induce macrophages to synthesize and release pro-inflammatory cytokines, including IL-1β via exosome-associated fibronectin (134). In contrast to the trophoblast, other tissues such as the prostate and placenta also secrete EVs to attenuate immune activity and facilitate normal physiological activity such as fertilization and pregnancy. It has been proposed that EVs in semen and secretion from placental explants mediate protection of the sperms and fetus from immune attack by the host tissues via NKG2D ligands, which reduce cytotoxicity of NK and CD8^+^ T cells (135, 136). EVs have also been implicated in pregnancy-associated immune suppression via the expression FasL, which is known to induce T cell anergy (137, 138). Other tissues that secrete EVs to attenuate inflammatory response include EVs secreted by intestinal epithelium to promote oral tolerance in rat (139) and EVs in milk and colostrum that exert immunosuppressive effects on T cells (140).

## Therapeutic Stem Cells and Exosomes

Mesenchymal stem cell, a widely distributed stromal support cell in body, is the most widely used stem cell type in clinical trials today with more than 306 trials^1^ to treat many diseases including many immune diseases such as multiple sclerosis (141) and auto-immune rheumatologic disorders (142). MSCs have been reported to exert both suppressive and regulatory effects on autologous and allogeneic adaptive and innate immune cells (143) such as inhibiting mitogen-activated T cell proliferation (144–148), inducing an anti-inflammatory tolerant phenotype in immune cells (149) and inhibit B cell proliferation (150). These effects provide a rationale for the approval of MSC in the treatment of pediatric graft-versus-host disease (GVHD) in Canada and New Zealand, and in its testing against immune diseases such as Crohn’s disease (151) and type 1 diabetes (152). However, the molecular mechanism underpinning these immune activities remains unresolved. It is observed that the efficacy of MSCs against severe GVHD (153) does not impair graft versus-leukemia (GVL) reaction (154) or suppress T cell proliferation (155). Increasingly, it was proposed that MSC secretes factors such as interferon-γ (IFNγ), TGF-β, PGE_2_, HLA-G, IL-10, and indoleamine 2,3-dioxygenase (IDO) to expand Tregs, which in turn, attenuate GVHD [reviewed in Ref. (156)]. However over the years, each of these secreted factors could not by themselves adequately account for the immunomodulatory activity of MSCs [reviewed in Ref. (157)]. For example, the immunomodulatory activity of MSCs was not compromised by a lack of IDO production caused by either defective IFN(γ) receptor 1 or IDO inhibitors (158) thus eliminating interferon-γ (IFNγ) (159–161) and IDO (162) as the secreted candidates for MSC immune activity.

The molecular mechanism underpinning MSC-mediated immunoregulation is likely to be complex and mediated by the synergism of several rather than single molecules. Hence, exosomes or EVs, which carry a multitude of molecules, are ideal secreted agents to mediate the immune-modulatory activity of MSCs. MSCs were first reported to secrete microvesicles in 2009 (163) and exosomes in 2010 (49). Some features of the immunomodulatory property of MSCs have been replicated by purified MSC exosomes. In a mouse model of myocardial ischemia/reperfusion injury, MSC exosomes attenuate injury induced inflammatory response as evidenced by reduced white blood cell count and tissue infiltration of neutrophils (164). We have also shown that MSC exosomes delay the rejection of allogeneic skin grafts in mice with a concomitant increase in Tregs (22) and inhibit complement-mediated lysis of sheep red blood cells in a CD59-dependent manner (165).

## EVs or Exosomes as Immunotherapeutic Agents

The extensive implication of EVs in modulating diverse aspects of the immune system to either enhance or suppress immune activity makes EVs attractive candidate immunotherapeutics. While this functional dichotomy increases the versatility of EVs as therapeutic agents, it also increases the risk of unpredictable adverse outcomes. To mitigate this risk, it is therefore critical that EVs with the appropriate functional properties are isolated for therapy. As discussed earlier, the capacity of EVs to enhance or suppress immune activity depends on the cell source, state of the cell source, e.g., maturity, and the type of EV. Hence a minimum requisite for the development of EVs as immunotherapeutic agents is a physical and biological characterization of each of the different EV types produced by a cell in a specific physiological state such that the EV type with appropriate functional properties can be isolated. Unfortunately, this is a highly intractable issue that is endemic in the EV research community. As discussed in the Section “Introduction,” there is presently a lack of clarity in defining the classes of EVs, the role of EVs or exosomes as mediators of intercellular communication. There is presently a concerted effort within the EV research community^2^ to first define and standardize the nomenclature for EVs and the EV isolation.

In spite of these challenges, the prospect of EV-based immunotherapy remains highly promising. Unlike cell-based immunotherapy, EV being a non-viable cellular product has many advantages. In cellular immunotherapy, the need to preserve cell viability increases a layer of complexity to its manufacture, storage, transport, and transplantation. In contrast, EVs are biologics and are thus more amenable to a strictly regulated and monitored manufacturing process. This will essentially translate into better qualified and safer off-the-shelf products that could be delivered to patients in a timely manner. The administration of EVs instead of cells would also alleviate many of the risks associated with viable cellular therapy. The use of viable replicating cells as therapeutic agents carries unique safety risks and challenges as the biological potency of the agent may persist or amplify even after the disease has been resolved, leading to adverse immune dysfunctions such as increased cancer risk and increased susceptibility to infection or auto-immune disease. In addition, the process of administering cells while generally safe (166) could cause complications such as occlusions in the distal microvasculature, as cells are generally much larger than the usual therapeutic small molecules or biologics (167). Furthermore, the differentiation potential of cells could generate inappropriate and potentially deleterious cell types. For example, the osteogenic and chondrogenic potential of MSC has raised safety concerns as a high frequency (51.2%) of ossifications and/or calcifications in tissues has been reported in some animal studies (168). In conclusion, the benefits of an EV-based immunotherapy vis-a-vis the risks of a cell-based immunotherapy provide a compelling rationale to develop an EV-based immunotherapy.

## Conflict of Interest Statement

The authors declare that the research was conducted in the absence of any commercial or financial relationships that could be construed as a potential conflict of interest.

## Footnotes

^1^http://www.clinicaltrials.gov/; accessed March 2013

^2^http://www.isev.org
